# The Premise of Intersectionality for Family Therapy Interventions With Asylum Seekers in the United States

**DOI:** 10.1111/jmft.70047

**Published:** 2025-06-22

**Authors:** Christian D. Chan, Chanel Shahnami Rodriguez, Connie T. Jones

**Affiliations:** ^1^ Department of Counseling and Educational Development The University of North Carolina at Greensboro Greensboro North Carolina USA; ^2^ School of Counseling Marymount University Arlington Virginia USA

**Keywords:** asylum seeker, cultural identity, family relationships, intersectionality, racist nativism

## Abstract

Changes in migration policy and governmental systems have increased anti‐immigration rhetoric and attitudes toward asylum seekers within the United States. Consequently, asylum‐seeking families contend with changes in culture, relationships, and roles, which exacerbate experiences of trauma, isolation, and mental health symptoms. While the United States still harbors an atmosphere of racist nativism, postmigration stressors uncover other forms of structural oppression, such as heterosexism and genderism. Intersectionality serves as an indispensable theoretical framework to examine intersecting forces of oppression and how they accentuate asylum‐seeking family experiences in therapy. To address sociopolitical experiences and oppression impacting the well‐being and relationships of asylum‐seeking families, the article (a) outlines key definitions and research trends on family relationships and interventions with asylum‐seeking families; (b) elaborates intersectionality's core tenets; and (c) synthesizes applications from intersectionality to enhance asylum‐seeking family interventions and research.

## 1 Introduction

Attention to migrant communities has gained further traction among researchers and family therapists in an effort to support transnational families through acculturation, separation, and displacement (Larrinaga‐Bidegain et al. 2024; Roy and Yumiseva 2021). Although migrant communities are vastly categorized into sojourners, immigrants, refugees, and asylum seekers, family researchers and therapists often conflate the experiences of these individuals rather than exploring distinct transitions, acculturation, and migration histories (Douglas et al. 2019; Graf et al. 2023). Refugees and asylum seekers, in particular, encounter forced migration that displaces them as a result of torture, war, violence, and persecution and create untenable living conditions within their country of origin (Bemak and Chung 2017; Utržan and Wieling 2020). Refugees and asylum seekers face markedly different trajectories when encountering legal, political, and social barriers that hinder and deny their access to therapy services and health care (Bemak and Chung 2017; Blount and Acquaye 2018). Despite the uptick of research on refugee experiences, especially in relation to trauma‐informed practices (see Bemak and Chung 2017; Flanagan et al. 2020; Midgett and Doumas 2016; Simmelink McCleary et al. 2020), less attention has been given to the sociopolitical context and lived experiences facing asylum seekers transitioning into the United States (Attia et al. 2023; Merry et al. 2017). More specifically, asylum seekers continue to face a multitude of psychosocial factors, torture, and trauma histories that influence their cultural identity, well‐being, and ability to seek help (Attia et al. 2022, 2023; Shaw and Verghese 2022). With distinct cultural experiences and histories, asylum seekers face unique types of precarity (Griswold et al. 2021). To this end, family therapy research could benefit from a more nuanced examination of cultural factors, sociopolitical contexts, and therapeutic interventions that zero in on asylum‐seeking families.

Illuminating the complex experiences of precarity facing asylum seekers, recent studies have outlined intervention trends to better address the needs of both refugees and asylum seekers. A study by Griswold et al. (2021) showed that asylum seekers contend with distinct forms of resettlement and postmigration stress, which alludes to the temporal implications of social context within families (Rivas‐Koehl et al. 2023). Aside from educational and linguistic challenges, asylum‐seeking families face various forms of traumatic stress (Flanagan et al. 2020; Gandham et al. 2021; Utržan and Wieling 2020), mental health issues (Báez et al. 2024; Patterson et al. 2018), and marked changes in family relationships and roles (Hadi 2023; Sigmund 2023; Wieling et al. 2020). Unfortunately, many of these adverse consequences are heightened by a compounded sense of ambiguity unique to asylum seekers (Utržan and Northwood 2017), given that many asylum‐seeking families within the United States are not afforded government services, protections, or timelines regarding their asylum status (Phillips 2023; Sigmund 2023).

Meanwhile, family studies and family therapy researchers have documented the significance of family‐based interventions for promoting cultural identity within racialized and migrant youth (Bámaca‐Colbert et al. 2019). As cultural identity development can serve as a protective factor and promote resilience intergenerationally across historically marginalized families (Perez‐Brena et al. 2024; Umaña‐Taylor and Hill 2020), promoting cultural identity and critical consciousness surrounding sociopolitical barriers strengthens the empowerment of historically marginalized families (Juang et al. 2017). Changes to family relationships and roles mired in migration stress are not without cultural and political implications (Larrinaga‐Bidegain et al. 2024; Roy and Yumiseva 2021; Wieling et al. 2020), given that many roles shift according to race, gender, and class (Attia et al. 2024; Sigmund 2023). In fact, Gangamma and Shipman (2018) discussed how transnational contexts introduce social and political influences that ultimately shape the expression of social identities, mainly in postmigration environments. Thus, integrating a comprehensive approach of cultural, social, and political context can be crucial to interventions with asylum‐seeking families.

Intersectionality—an analytical framework for examining multiple overlapping forms of oppression and power inequities—encompasses many of these facets by linking racism, ethnocentrism, and nativism together while explicitly demystifying the contextual factors that harm asylum seekers (Bonu Rosenkranz 2024; Collins and Bilge 2020). Due to intersectionality's grounding in a social justice ethos (Collins 2015, 2019), explicit incorporation of intersecting identities and forms of oppression (Few‐Demo 2014; Few‐Demo and Allen 2020), and a conceptualization of systemic and legal barriers (Cho 2013; Cho et al. 2013), the approach can serve as an intuitive application for family therapists and researchers to raise important areas of cultural factors and power relations within asylum seeker families (Attia et al. 2023; Sigmund 2023). To accentuate family interventions through intersectionality, this article (a) outlines key definitions and research on family relationships and interventions with asylum‐seeking families; (b) elaborates on philosophical underpinnings of intersectionality; and (c) illustrates applications for family therapists and researchers to employ intersectionality with asylum‐seeking families.

## 2 Asylum Seekers

Asylum seekers are a group of individuals that are searching for a new, permanent place to live, work, and find protection without well‐founded fear, yet do not have the same legal recognition as refugees (Amnesty International, n.d.; Phillips 2023). Historically, there has been a tenuous atmosphere surrounding US immigration reform that dictates policies for individuals entering the United States (Alberto and Chilton 2019; King 2022). Anti‐immigration rhetoric and policies are shrouded in racist nativism—“the native person's right to dominance is justified by assigning values to real or imagined differences; natives are perceived to be white, while non‐natives are perceived to be people of color” (Risley 2022, p. 113). Recently, the United States has made the process of seeking asylum more arduous with policies that have attempted to dissuade families from migrating to the United States (Alberto and Chilton 2019). Xenophobic policies, such as the Zero Tolerance Policy implemented in 2018, the Migrant Protection Protocols (MPP), and the Asylum Transit Ban, affect asylum‐seeking legal processes and exacerbating traumatic experiences that asylum seekers have already faced before arriving to the United States (Alberto and Chilton 2019; American Immigration Council 2023a). The United States has been consistently criticized for their discrepancies, loopholes, and biases in policy and implementation with refugees and asylum seekers (King 2022). More recently, the U.S. Committee for Refugees and Immigrants (USCRI; 2025) denounced the U.S. Department of Homeland Security for revoking humanitarian parole protections to Venezuelans, Cubans, Haitians, and Nicaraguans, who have received temporary protections for legal entry to the United States.

Despite overlaps between asylum seekers and refugees, discrepancies still remain in how their statuses are uniquely defined. According to the United Nations Convention Relating to the Status of Refugees 1951, the United Nations High Commissioner for Refugees created a shared definition for asylum seekers and refugees that still currently stands today as: “owing to a well‐founded fear of being persecuted for reasons of race, religions, nationality, membership of a particular social group or political opinion, is outside the country of their nationality and is unable or, owing to such fear, is unwilling to avail themself of the protection of that country” (Article 1A, para. 2). Utržan and Northwood (2017) described that the *well‐founded fear* as past or future persecution can be directly a result of acculturative stressors, missing and separated family members, or national violence in country of origin. Despite these worldwide strides to legally define and support asylum seekers and refugees, asylum seekers navigate differing political circumstances that do not afford the same protections (Douglas et al. 2019; King 2022). The UN Geneva Convention definition covers the reasons for seeking refuge or asylum, but does not dictate recommendations and procedures for processes after arrival to a country nor the length of time allowed for asylum. For an individual to seek asylum, they need to physically be inside the United States or at a US port of entry (American Immigration Council 2023b). Generally, individuals must apply for asylum within 1 year of arriving in the United States (American Immigration Council 2025), unlike refugees that typically wait at a refugee camp while being screened extensively for about 1 to 1.5 years and are provided transportation from the US government to migrate to the United States (Bemak and Chung 2015).

Research on asylum seekers suggest growing trends of mental health inequities and symptoms. Due to mental health and traumatic stress on asylum seekers and their families (Merry et al. 2017), mixed‐status families with US citizen children and immigrant parents have more symptoms of PTSD, anxiety, and depression, partly due to the potential risk of detained or deported parents (Simmelink McCleary et al. 2020; Wieling et al. 2020). According to a systematic review by Flanagan et al. (2020), trauma exposure among refugee and asylum‐seeking parents yields mental health issues, disrupted attachment, maladaptive parenting, and diminished family function, which result in unfavorable mental health outcomes among children. Of note, many studies in their systematic review rarely distinguished asylum‐seeking and refugee participants in family research, which can obscure some of the demands associated with asylum‐seeking parents, youth, or families. Another systematic review by Mak and Wieling (2022) showed that many available interventions to support displaced and trauma‐affected communities are largely focused on symptom reduction rather than any form of relational interventions (e.g., family, parent‐child, couple). A more recent study by Attia et al. (2024) documented a litany of stressors related to migration trauma, including inhibited access to resources, isolation, ongoing stress with the asylum process, and constant fears of deportation. These issues coincide with recent trends of detention of female asylum seekers, including sexual and physical abuse in US detention centers (Hadi 2023). According to a qualitative study by Singer et al. (2023), COVID‐19 exacerbated underlying conditions of anxiety, depression, and posttraumatic stress disorder among asylum seekers in the United States; in fact, most of these symptoms are combined with legal barriers to health care and discrimination associated with asylum status. Findings from the Griswold et al. (2021) study reinforced these overlaps, as participants reported a litany of unmet mental health needs while waiting on approvals for work, healthcare access, and basic needs of food security and housing.

## 3 Asylum‐Seeking Families

Separation from family represents a significant barrier to positive psychological outcomes in asylum seekers (Larrinaga‐Bidegain et al. 2024; Santilli et al. 2023). Consistent with transnational migration, issues of separation, culture shock, and acculturation issues can rupture family unity (Bámaca‐Colbert et al. 2019; Miller and Csizmadia 2022). Consequently, parents and adult caregivers become more dependent on children due to children acculturating faster than adults, where children are sometimes used by their parents, other caregivers, and service providers to serve as cultural brokers (Blount and Acquaye 2018; Perez‐Brena et al. 2024). During resettlement, families often serve as crucial sources of connection (Utržan and Northwood 2017; Utržan and Wieling 2020), yet parents can struggle with meeting the complex needs of their children due to other occupational, health care, and housing stressors (Báez et al. 2024; Merry et al. 2017). In fact, an ethnographic study by Sigmund (2023) illustrated that racial and legal barriers heightened intersecting forms of precarity for children and family members, which in turn, affected Central American asylum‐seeking mothers' ability to be emotionally available. Another study by Parra‐Cardona et al. (2017) reinforced the need to integrate culture and targeted parenting interventions to address discrimination, which could better support child mental health outcomes. Additionally, Parra‐Cardona et al. (2017) showed that gender differences, even in the presence of culturally adapted interventions, shape parenting interventions for child mental health and require unique intersecting nuances of discrimination.

Changes in family roles can be especially difficult to adjust to and may adversely affect psychological adjustment in post‐migration (Flanagan et al. 2020). The parental mental health status, particularly the emotional well‐being of the parent, and stable familial relationships seem to be crucial to psychosocial development and protective factors against trauma (Merry et al. 2017; Parra‐Cardona et al. 2019). Children may lose dependency on their parents and other adult caregivers as they witness a transformation from autonomous caretakers to overwhelmed and dependent individuals who are trying to learn a new language and customs while trying to serve as the provider for the family (Bemak and Chung 2015, 2017; Miller and Csizmadia 2022). For example, Utržan and Wieling's (2020) study described how post‐resettlement experiences for adult refugees were still riddled with guilt in relying on others as language brokers when not speaking much English. Findings from Simmelink McCleary et al. (2020) point to hesitations about discussing emotional well‐being due to the lack of emotional literacy or stigma around mental health, which underscore gaps in communication about post‐migration stress in families.

Despite post‐migration stressors, participants in the Griswold et al. (2021) study highlighted the importance of family interventions as a way to reconnect with community and address their mental health needs. In another study by Santilli et al. (2023), some asylum seekers identified that they found courage through reconnecting with their family in a new country. Family interventions seem to be crucial within post‐migration experiences, as Parra‐Cardona et al. (2019) showed that addressing discrimination and family problem‐solving can produce helpful outcomes for family members' mental health. While asylum seekers are broadly lodged in legal and political systems of power, post‐migration experiences can specifically challenge asylum‐seeking family members' relationships by forcing them to renegotiate their cultural values and statuses of power (Báez et al. 2024).

## 4 Intersectionality Theory as a Foundation for Practice With Asylum Seekers

Relevant to the sociopolitical context facing asylum‐seeking families (Gangamma and Shipman 2018; Interiano‐Shiverdecker et al. 2022), intersectionality theory has spanned multiple decades of research that analyzes key issues of culture, identity, and policy facing multiply marginalized communities (Cole 2020; Curtis et al. 2020). Coined by Crenshaw (1989) in solidarity with critical legal scholars, intersectionality underscores the examination of power, privilege, oppression across myriad legal outlets, mental health professions, and social science disciplines through locating structural forms of oppression (see Bonu Rosenkrantz 2024; Chan et al. 2018; Few‐Demo 2014; Ratts et al. 2016; Singh et al. 2020). Intersectionality posits that structural forms of oppression intersect, thereby eliciting more complex inequities (Bowleg 2017) while generating clinical interventions that address how multiply marginalized individuals experience harms of power, suppression of culture, and further exclusion (Buchanan and Wiklund 2020).

Family studies and family therapy researchers have taken a vested interest in using intersectionality to generate more intricate analyses of intersecting forces of oppression, such as racism, heterosexism, and genderism, embedded within familial dynamics and societal pressures (Few‐Demo and Allen 2020; Fish et al. 2024; Gangamma and Shipman 2018; Reczek 2020; Seedall et al. 2014). Aside from documenting the complexities of social identity underlying family relationships, intersectionality reveals connections between identity in families in tandem with power structures, temporal implications, and social context (Rivas‐Koehl et al. 2023). Despite its uptick in family research, Curtis et al. (2020) reported that intersectionality remains scarce in empirical and clinical applications within family science and family therapy and lacks a consensus on operational definitions. Despite more recent applications of intersectionality in transnational contexts (e.g., Gangamma and Shipman 2018; Interiano‐Shiverdecker et al. 2022), these references overlook more precise applications for asylum seekers and warrant a more substantial explication in conceptual and empirical research (Attia et al. 2022, 2023).

Often, a multitude of scholars frequently define intersectionality only as multiple identities or diverse identities (Bowleg 2008; Moradi and Grzanka 2017), yet omit intersectionality's commitment to a clear social justice ethos (Bilge 2013; Collins and Bilge 2020); a genealogy linked to Black feminism, women of color, and queer women of color (Bonu Rosenkrantz 2024; Cole 2020); and an analysis of power relations (Bowleg and Bauer 2016; Grzanka 2020). The impact of intersectionality in practice is usually reduced as a result of scholars and practitioners distancing the theory from its clear connection to power and structures (e.g., schools, communities, policies; Bowleg 2017; Curtis et al. 2020). Additionally, several scholars (e.g., Chan and Howard 2020; Grzanka 2020; Hancock 2016; Moradi and Grzanka 2017) continue to implore researchers and practitioners to root the practice of intersectionality in histories of women of color and queer women of color who contributed to its evolution. Otherwise, intersectionality remains a diluted framework that distorts its underpinnings and genealogy in race and politics (Buchanan and Wiklund 2020; Collins 2015). As intersectionality becomes a standard practice for family therapists and researchers, it is imperative to incorporate the influences of Crenshaw (1989, 1991); Collins (1986); Davis (1983); Lorde (1984); Anzaldúa (1987); Combahee River Collective (1995); hooks (1981); and Moraga and Anzaldúa (1983) for the critical thinking connected to culture, identity, politics, and social justice.

### 4.1 Core Underpinnings of Intersectionality

According to Collins and Bilge (2020), intersectionality focuses an analysis on six areas: (a) social justice; (b) relationality; (c) complexity; (d) social inequality; (e) power; and (f) social context. Intersectionality operates primarily from a foundation in *social justice*, where family therapists and researchers are tasked with critiquing structural inequities and creating systematic steps to dismantle these inequities (Few‐Demo 2014; Singh et al. 2020). Invoking intersectionality is not merely a description of inequitable systems or a revelation of the phenomena facing multiply marginalized groups, but rather a strategy to denounce hegemonic social and power structures (Buchanan and Wiklund 2021; Matsuda 1991). Relevant to asylum‐seeking families, simply mentioning cultural or political factors underlying their therapy experience may not be sufficient for their well‐being. Rather, policy‐based interventions (Alberto and Chilton 2019; Cole and Duncan 2023) and addressing social determinants of racism, nativism, and classism that inhibit safety and consistent participation in family therapy (Interiano‐Shiverdecker et al. 2022; Rivas‐Koehl et al. 2023) could be more crucial.

Intersectionality also highlights the importance of *relationality*, where family therapists and researchers interconnect the relationships across multiple dimensions of social identities (e.g., race, ethnicity, sexuality, affectional identity, gender identity, ability status, spirituality; Chan and Howard 2020). These relationships mirror the connections among multiple overlapping forms of oppression (e.g., racism, genderism, classism) that result in barriers, isolation, and marginalization from society (Curtis et al. 2020; Few‐Demo 2014). To consider this aspect in family studies and therapy, family therapists and researchers can conceptualize the effects linked across these overlapping forces of oppression and identify how specific communities (e.g., queer and trans people of Color) face oppression within asylum‐seeking communities (Corlett and Mavin 2014; Parent et al. 2013). Attia et al. (2022), for instance, considered how LGBTQ+ asylum seekers face compounded levels of post‐migration stress not only as a function of racism and nativism within the United States but also heterosexism that existed both in the United States and their country of origin.

A third component of intersectionality relates to *complexity* by highlighting the numerous dimensions of cultural and social identities and recognizing that each of these comprises unique experiences (Carastathis 2014, 2016; Collins 2015). For instance, asylum‐seeking families could consist of LGBTQ+ parents, diverse linguistic experiences, gender identities, mixed‐status parents and children (e.g., US born children to asylum‐seeking and undocumented parents), and different racial and ethnic backgrounds and impact their overall family culture and functioning (Interiano‐Shiverdecker et al. 2022). These backgrounds and identities are not essentialized, which means that not all asylum seekers will carry the same experiences (Chan et al. 2018). Consequently, complexity infers that individuals with these multiple dimensions can carry multiple forms of marginalization or simultaneously experience privilege and oppression (Gangamma and Shipman 2018; Warner et al. 2016).

*Social inequality* is a fourth theme involved in the framework of intersectionality (Collins and Bilge 2020). Responsibly employing intersectionality requires family therapists and researchers to examine how asylum seekers continue to face a myriad of legal and political barriers that culminate in their displacement and isolation from society (Attia et al. 2022, 2023). Through intersectionality, family therapists and researchers are tasked with recognizing these social inequities as ways in which asylum seekers can become disconnected from crucial resources, education, and access to mental health and counseling services due to their legal status and US policy constraints (Alberto and Chilton 2019; Sigmund 2023). Unique to asylum seekers and family therapy, the lack of refugee status forecloses employment and healthcare opportunities and compounds unique forms of inequity due to ambiguous placement, length of time, and legal repercussions (King 2022). Given this lens on social inequity, family therapists and researchers must consider a fifth key aspect of intersectionality with *power*. Relevant to social inequity, power is an underlying factor for characterizing intersectionality (Cole 2020; Grzanka 2020). At the crux of the framework, analyzing power posits that practitioners and researchers must recognize which communities may not have access to resources, may not have influence on policy, may not be seen as the norm, and may be excluded from important community, legislative, and policy‐based decisions (Bonu Rosenkrantz 2024; Few‐Demo et al. 2016). Through analyzing power relations, practitioners and researchers can detect interpersonal interactions, policies, and cultural norms that can disempower multiple communities of asylum seekers (Cole and Duncan 2023; Gangamma and Shipman 2018). Distinct within family therapy practice, therapists must consider the power context within families when structural forms of oppression (e.g., racism, nativism) enact certain power relations between family members (Few‐Demo et al. 2016; Rivas‐Koehl et al. 2023). For instance, queer and trans asylum‐seeking parents could encounter more deleterious forms of prejudice outside of their family from school and legal systems while managing potential biases from heterosexual children. In mixed‐status families, asylum‐seeking families could face a marked shift in levels of acculturation with children learning more linguistic and cultural norms more quickly and shifting the power balance as cultural brokers within the family (Mak and Wieling 2022; Miller and Csizmadia 2022). Power becomes much more nuanced in asylum‐seeking families in redefining the internal power dynamics of the family and altering traditional parenting roles and styles (Utržan and Northwood 2017). Reflecting on social inequity and power, *social context* is the final core tenet comprising the intersectionality approach (Collins and Bilge 2020). Social context provides a map for organizing power relations by explaining which groups may have more power and access in a certain environment due to the surrounding policy, representation, or politics (McKinzie and Richards 2019; Rivas‐Koehl et al. 2023). Additionally, social context alludes to the background that positions certain groups with factors of privilege and other historically marginalized groups in oppression (Carastathis 2016; Collins and Bilge 2020). To this end, social context is crucial in discerning how policy, legal, and healthcare implications influence power relations within the family, often resulting in uncertain shifts within roles and generational norms (Sigmund 2023).

## 5 Implications for Clinical Practice

Principles of intersectionality have become a more viable framework as a way to honor complex power dynamics and political connotations impacting families. Given that family researchers and therapists commonly examine systems, socialization process, and ecology, they possess the assets and skills to translate intersectionality effectively into practice (Fish et al. 2024; Rivas‐Koehl et al. 2023). Infusing intersectionality into family practice specifically with asylum‐seeking families can be beneficial as they navigate shifting social contexts, manage shifts in their identity, and respond to encounters of oppression (e.g., Zero Tolerance Policy, limited protections in the United States).

### 5.1 Examining Intersecting Inequities as Barriers to Family Therapy

Family therapists have an opportunity to assess for intersecting forms of inequity that prevent asylum‐seeking families from consistently engaging in the process of family therapy (Griswold et al. 2021). Due to the compounding toll of extreme stress on asylum‐seeking parents, it is likely that many asylum‐seeking families refrain from seeking mental health support for their family or children (Patterson et al. 2018). While navigating legal, political, and healthcare systems within the United States, asylum‐seeking families may be attending to other critical survival needs (e.g., housing, medical care, food security) that could make family therapy a lesser priority, despite unmet mental health needs (Utržan and Northwood 2017). Additionally, asylum‐seeking families may be focusing on managing other needs, such as steady employment, to maintain access to legal resources and community support (Sigmund 2023).

Rather than assuming parental deficits or resistance to family therapy, intersectionality elicits a more critical understanding of structural forms of oppression that accentuate how family members might present themselves in the family therapy context (Gangamma and Shipman 2018). To this end, intersectionality can be generative by examining intersecting inequities of asylum‐seeking families. For instance, family therapists can consider how the nexus of nativism and racism promotes linguistic forms of marginalization that further limits opportunities in employment, health care, education, and housing and reduces access to available family therapy providers who speak their language (Bunn et al. 2022). Additionally, family therapists can leverage intersectionality by considering how other forms of oppression underlie nativism and racism. For example, queer and trans asylum‐seeking parents may be subject to housing discrimination due to pervasive biases in housing placements based on gender expression, sexual and gender minority intimate relationships, and ambiguity on length of stay (Attia et al. 2022). In fear of repercussions from housing, they may be hesitant to engage in family therapy by potentially disclosing salient identities or legal information that could threaten their asylum status (Attia et al. 2024; Interiano‐Shiverdecker et al. 2022).

Family therapists could explore how critical instances of discrimination could undermine opportunities for financial support and livable wages (Santilli et al. 2023). In addition, the combination of racism, genderism, and transnationalism may exacerbate inequitable treatment toward asylum‐seeking mothers whose opportunities remain limited due to legal barriers, linguistic requirements, and gendered perceptions of work (Phipps et al. 2022; Sigmund 2023). Because family therapists can be likened to authorities from their countries of origin, it is important for family therapists to reduce healthcare stigma and service hesitancy by explaining their role, including how their role will not have repercussions on their asylum process (Báez et al. 2024; Reading and Rubin 2011; Singer et al. 2023). In family therapists' assessment of inequities, family therapists take into account a transnational perspective by examining legal and political barriers toward health care, housing, and asylum status in the United States and their respective countries of origin (Attia et al. 2022, 2024).

### 5.2 Managing Power Relations Between Family Members

Related to power and social inequality in asylum seeker families, family therapists possess a unique opportunity to identify relational dynamics occurring collectively and between family members. Given the premise of differing social identities across family members, therapists could examine various interpersonal interactions between family members to determine if social inequalities are replicating within the family (Few‐Demo 2014; Gangamma and Shipman 2018; Seedall et al. 2014). For instance, an asylum seeker youth who is queer may be silenced by a parent who harbors homophobic and transphobic prejudices as a result of stigma from the culture in their country of origin (Attia et al. 2022). Due to intersecting of social class, financial, and legal implications that could threaten their asylum status (Sigmund 2023; Utržan and Wieling 2020), the youth member of the family may not wish to challenge their parent or disclose their queer identity, yet desire support (Attia et al. 2022). Rather than portraying a fault of parenting, family therapists could leverage intersectionality to explain how particular identities, such as queerness and gender nonconformity, surface differently within the United States and explain the parents' socialization from a homophobic and transphobic culture in the country of origin (Gangamma and Shipman 2018). Based on a compounded fear of consequences (Utržan and Wieling 2020), parents may generalize the stigma and persecution from their own countries of origin and apply it to the postmigration context of their children's identities (Simmelink McCleary et al. 2020). Similarly, the therapist could facilitate opportunities for discussions on the differences between power, privilege, and oppression based on their identities, namely, how an experience of nativism and racism can instigate fears similar to heterosexism (Chan 2018). By resolving conflicts related to power, privilege, and oppression within an asylum‐seeking family, the therapist may be able to promote support and cohesion among family members, which they may need to buffer the effects of acculturation and displacement (Roy and Yumiseva 2021; Yzaguirre and Holtrop 2025). Integrating the tenet of social justice is not only referring to the therapist's ability to facilitate empowerment among asylum seeker families. Social justice requires an intentional effort on the practitioner to consider forms of advocacy and policies that may be obstructing the protections of asylum‐seeking families or creating further displacement in their connection to a specific environment (Curtis et al. 2020; Rivas‐Koehl et al. 2023; Wieling et al. 2020).

### 5.3 Reflexivity in Therapist‐Family Relationship

Although intersectionality can serve as a foundation for practice, there are considerations that underlie its use in family therapy and the social location as family therapists. Therapists may struggle with accurately employing intersectionality's principles, where they can unfortunately reduce the entire approach to multiple identities and diverse identities rather than grasping intersectionality's social justice ethos and attention to structural oppression (Bilge 2013; Grzanka 2020; Moradi and Grzanka 2017). In some cases, therapists may be retreating into one tenet of intersectionality or depoliticizing their practice to assuage their own discomfort with internalized oppression or discussions of political implications facing asylum seekers (Buchanan and Wiklund 2021). As an unfortunate scenario, some therapists may ignore postmigration factors affecting asylum status and inadvertently eschew acknowledging experiences of racism, nativism, and classism (Gangamma and Shipman 2018; Interiano‐Shiverdecker et al. 2022). Even family therapists with a refugee background should understand how key differences in their migration journey could tremendously differ with other asylum‐seeking family members. As therapists prepare to work with asylum‐seeking families, they can explore their own social identities through internal reflections, along with explicit conversations during supervision and consultation.

A number of therapists leverage intersectionality primarily from the lens of individual counseling modalities (e.g., therapist‐client) rather than suggesting clear ideas to implement in family therapy practice (Chan 2018). Therapists using family therapy modalities are required to wrestle with the complexity of social identities (e.g., race, gender identity, ethnicity, migration status) with multiple family members (Abreu et al. 2020; Chan and Erby 2018). They must be adept at identifying which social identities may be salient to asylum‐seeking families and which social identities may be invisible (Attia et al. 2022). To be intentional with intersectionality in family therapy requires an in‐depth level of reflexivity to offset the possibility of overlooking notable social identities, mistiming discussions of oppression, or retraumatizing family clients (Gangamma and Shipman 2018; Patterson et al. 2018). To this end, therapists need to be mindful of systemic forms of injustice impacting the transnational experience and interrogate their own biases on asylum‐seeking families, including a lack of knowledge of key political issues and policies (Wieling et al. 2020). Aside from simply managing differing identities that organize privilege and oppression between therapists and specific family members, therapists can examine how their own identities influence their beliefs around other cultural beliefs, such as religious beliefs and gendered parenting practices, which shape the focus of discussions in family therapy. In examining their own beliefs, they can preemptively ensure that they do not stigmatize asylum‐seeking family members' experiences, especially in cases where the therapist shares a privileged identity with another family member (e.g., male, cisgender, white, heterosexual, nondisabled).

### 5.4 Harnessing Strengths of Complexity and Dynamic Cultural Shifts

By specifically employing the tenet of complexity, therapists could introduce opportunities in which asylum seeker family members hold multiple identities that relay diverse critical vantage points and solutions (Attia et al. 2023). Relevant to asylum‐seeking families, it may be beneficial to consider a multitude of social identities, including race, ethnicity, nationality, social class, sexuality, and gender identity, that coincide with their status as asylum seekers (Gangamma and Shipman 2018). Exploring the diversity of these social identities can hold profound meaning for each family member as they construct new meaning through the legal process and acculturation to a new host culture (Sigmund 2023). For example, youth in asylum‐seeking families may be more attuned and aware of the critical needs of their families and parents when seeking asylum but need further support from therapists to facilitate communication (Simmelink McCleary et al. 2020). Highlighting the effectiveness of how an asylum seeker family navigates diverse vantage points across social identities related to their experience of racism and nativism can elicit opportunities for empowerment.

Similarly, relationality can play a substantial role in practice by identifying points in which family members navigated multiple overlapping forms of oppression in their migration history. For instance, asylum‐seeking parents might develop multiple perspectives on gender roles and expand their viewpoints after navigating genderism within two countries with their children (Yzaguirre and Holtrop 2025). This approach can show how intersectionality bridges certain identities to identify strategies or narratives in which asylum‐seeking families have overcome multiple forms of oppression (Attia et al. 2024).

### 5.5 Advocacy‐Focused Interventions

Employing intersectionality in family therapy to facilitate support and well‐being for asylum‐seeking families requires an ongoing commitment to advocacy. Given that intersectionality is anchored in the tenet of social justice (Collins and Bilge 2020; Curtis et al. 2020), family therapists can advance their interventions by harnessing opportunities for advocacy that move beyond mere critiques of power relations and social inequities (Rivas‐Koehl et al. 2023). Rather than waiting for asylum‐seeking families to present concerns and inequities in family therapy sessions, family therapists could take a more proactive approach by analyzing the impact of policies on asylum‐seeking family members' mental health and family relationships (Cole and Duncan 2023). To this end, family therapists could stay attuned to US policies that dictate asylum seekers' rights to economic, housing, and healthcare supports (Alberto and Chilton 2019; Phipps et al. 2022). Additionally, it could be beneficial for family therapists to form relationships with advocacy organizations that specialize in legal defenses and policy advocacy, such as the Asylum Seeker Advocacy Project (ASAP) or Charlotte Center for Legal Advocacy. In doing so, family therapists could play a crucial role in community‐organizing efforts that garner attention toward human rights violations and directly address larger levels of policy (Phillips 2023). Participating in such efforts can also illuminate more in‐depth complexities, such as human rights violations targeting reproductive justice (Hadi 2023) and economic supports for asylum‐seeking mothers (Sigmund 2023) that could impact their parenting practices.

Infusing the tenet of social justice in intersectionality recognizes that family therapy is not the sole intervention for health and safety within the United States (Gangamma and Shipman 2018). For some asylum‐seeking family members, such as children who migrate, they gain a wealth of navigational strategies and could be empowered by participating in advocacy initiatives and groups (Delgado 2022; Santilli et al. 2023). By partnering with local refugee, immigration, and asylum advocacy organizations, family therapists could host programming and multi‐family groups that connect asylum‐seeking families with support networks (Reading and Rubin 2011). For instance, programming and workshops could directly address safe approaches to navigate healthcare resources and leverage peer input from asylum‐seeking family members present at such events (Frounfelker et al. 2021; Singer et al. 2023). Harnessing partnerships with community organizations, family therapists could also develop initiatives that collect data on safe housing and employment resources directly from asylum‐seeking families. Leveraging the empowerment of youth in the community, they could also introduce possibilities for asylum‐seeking children to share strategies in which they assisted parents with navigating US legal systems (Delgado 2022; Parra‐Cardona et al. 2019). Engaging in advocacy partnerships with asylum‐seeking family members could also assist with destigmatizing the process of family therapy, thereby promoting its benefits and reducing stigma (Griswold et al. 2021).

## 6 Conclusion

Intersectionality serves as a relevant framework to identify the collective needs of asylum‐seeking families. It is a critical framework that expands the theoretical implications for development of asylum seekers and their definitions and relationships with family. Using intersectionality in support of asylum‐seeking families promotes opportunities to expand the theoretical application of intersectionality by acknowledging the temporal, legal, social, and political implications of asylum status (Attia et al. 2024; Rivas‐Koehl et al. 2023). Due to the compounded experience of racism and nativism, asylum‐seeking families encounter increased rates of traumatic stress and health disparities that shape their well‐being, identity development, and long‐term connection to family (Merry et al. 2017; Utržan and Northwood 2017). This framework underscores particular subgroups within families, such as asylum‐seeking mothers who may be navigating significant role changes based on racism, nativism, xenophobia, and genderism (Sigmund 2023). Intersectionality not only brings forth crucial opportunities to understand cultural nuances and identity development of asylum‐seeking families but also offers an analysis of structural violence within racist and nativist immigration policies (Alberto and Chilton 2019). Given intersectionality's premise, family therapists should consider multiply marginalized groups (e.g., LGBTQ+ asylum‐seeking youth and their family relationships; conflicts in parent‐child dyads with LGBTQ+ asylum‐seeking youth; asylum‐seeking youth and homelessness) in future research to further expand family therapy interventions. Family therapists can also encourage intersectionality within their training and supervision as opportunities to examine the social context and garner more critical attention to policies that harm family relationships and asylum seekers' mental health. By leveraging implications for clinical practice, family therapists can further expand on applications of intersectionality beyond practice to teaching, supervision, and advocacy.
